# Social inequality in mental disorder diagnoses and psychotropic medication use among 15-year-old adolescents in Denmark from 2002–2022

**DOI:** 10.1007/s00127-025-02943-y

**Published:** 2025-07-02

**Authors:** C. L. B. Sørensen, O. Plana-Ripoll, U. Bültmann, T. N. Winding, P. B. Steen, K. Biering

**Affiliations:** 1 Department of Occupational and Environmental Medicine, Gødstrup Hospital, Herning, Denmark; 2 Department of Clinical Epidemiology, Aarhus University and Aarhus University Hospital, Aarhus, Denmark; 3 National Centre for Register-based Research, Aarhus University, Aarhus, Denmark; 4 Department of Health Sciences, Community & Occupational Medicine, University Medical Center Groningen University of Groningen, Groningen, Netherlands

**Keywords:** Mental health, Adolescence, Equality, Socioeconomic status, Social inequality

## Abstract

**Purpose:**

The aim of this study is to examine if the social inequality in adolescent mental health has changed in the past decades (2002–2022) by studying the associations between socioeconomic status (SES) and mental health measures in 15-year-old adolescents.

**Methods:**

This study is a register-based study consisting of seven cross-sectional analyses of associations between adolescents’ SES, defined as family income and parents’ educational level, and mental health, defined as mental disorder diagnosis and medication use. The population consists of all registered residents in Denmark who turned 15 years in the years 2002–2022. All data was obtained from Danish population-based registers. The prevalence of mental health measures was calculated, and the associations between SES and mental health were analysed with log-binomial regression.

**Results:**

The prevalence of mental disorder diagnoses and medication use of adolescents increased during the past two decades. Associations between SES and mental health were found between all measures during the period, however, a trend toward decreasing associations for low-SES groups and stable odds ratios for high-SES groups compared to the middle-SES were observed. Diagnosis-specific analyses—including eight diagnostic categories—revealed divergent trends, such as increasing associations for SES and substance use disorders and decreasing associations for SES and mood disorders.

**Conclusion:**

This study highlights persistent but evolving social inequalities in adolescent mental health in Denmark from 2002 to 2022. While the prevalence of mental health diagnoses increased, changes in inequality patterns were diagnosis-specific, suggesting that broader societal trends may influence types of mental disorders differently.

**Supplementary Information:**

The online version contains supplementary material available at 10.1007/s00127-025-02943-y.

## Background

Poor mental health is an increasing global concern, with the World Health Organization (WHO) estimating that approximately 20% of children and adolescents are affected by a mental health condition [1]. Denmark has seen similar trends over recent decades, as reflected by an increase in both the prevalence of mental disorder diagnoses and medication use among youth [2–4]. Notably, antidepressant use among 0–17-year-olds in Denmark more than doubled the past decades, from 2.15 users per 1,000 inhabitants in 2002 to 5.04 users per 1,000 in 2022 [5]. This increase has occurred despite a regulatory change in 2014 that restricted general practitioners from prescribing antidepressant medication to children and adolescents [6]. The increased medication use aligns with rising incidence rates of diagnosed mental disorders in 15–20-year-olds; for example, a study found that the incidence rates of mood disorders in younger ages (under age 30 years) were markedly higher in the most recent birth cohorts compared to older cohorts [7]. Several studies on both self-reported mental health and mental disorder diagnoses have confirmed this increasing trend over time, while studies on psychotropic medication use are lacking [3, 4, 7, 8].

Rising mental health issues have paralleled an increase in socioeconomic inequality in Denmark. The Gini coefficient, reflecting income distribution, rose from 24 in 2002 to 30 in 2022 [9]. Moreover, social mobility through education has also become more difficult: in 2021, 60% of children from the poorest quintile who lacked vocational training remained in the poorest quintile, compared to 39% in 1995 [10]. The association between low socioeconomic status (SES) and poor mental health in adolescents is well-documented [11–17]. This has been evident, both when SES is defined as income, reflecting the economic resources of the family, and parents’ educational level, reflecting the cognitive skills and cultural capital [18]. However, it is also evident that the associations between SES and poor mental health differs depending on the measure of SES [11]. Given the importance of understanding the different aspects of SES in relation to adolescent mental health, further investigation is crucial. While existing studies tend to focus on specific aspects of SES, such as income or educational level, few incorporate population-based data [11–17]. Therefore, research that explores multiple SES factors within the same population-based sample remains limited. Moreover, although social inequality and adolescent mental health issues have increased simultaneously, little is known about the changes over time in the association between SES and mental health in adolescents.

This study aims to examine the association between social inequality in mental health using various SES measures (family income and parental educational level) and mental health measures (mental disorder diagnoses and medication use) in 15-year-old adolescents over the period from 2002 to 2022.

## Methods

### Study design, setting, and participants

This study is a register-based cohort study consisting of cross-sectional analyses of the associations between adolescents’ SES, defined as family income and parents’ educational level, and mental health, defined as mental disorder diagnoses and medication use, for seven 3-year-periods in the years 2002–2022.

The population consists of all registered residents in Denmark who turned 15 years in the years 2002–2022 identified through the Danish Civil Registration System [19].

### Variables, data sources and measurement

#### Mental health measures

We defined mental disorder diagnoses by the Danish modification of the 10th version of the International Classification of Diseases (ICD-10) codes F10-F69 and F80-F99, thus excluding F00-F09 (organic diagnosis) and F70-79 (intellectual disabilities). The mental disorder diagnoses are presented in 8 diagnostic group: substance use disorders (F10-F19); schizophrenia, schizotypal, and delusional disorders (F20-29)—hereafter referred to as “psychotic disorders”; mood disorders (F30-39); neurotic, stress-related, and somatoform disorders (F40-48)—hereafter referred to as “anxiety-related disorders”; eating disorders (F50-59); personality disorders (F60-69); developmental disorders (F80-89); behavioural disorders (F90-98); and a joint category of “Any mental disorder”. Mental disorder diagnoses from the psychiatric and somatic units from 1995–2022 are identified from the Danish National Patient Registry [20, 21]. The register does not cover outpatients before 1995 and therefore, the first cohorts of adolescents in 2002–2009 do not have complete data from birth and the results are not directly comparable with the later cohorts. Adolescents' mental disorder diagnoses are defined as the lifetime prevalence of any primary or secondary diagnosis recorded from birth until six months after the adolescent’s 15th birthday.

Medication use was defined as prescriptions for psychotropic medication using Anatomical Therapeutic Chemical (ATC) codes N05A (excluding N05AN), N05AN, N05B, N05C, N06A, N06B, N06C (excluding N06AX01 and N06AX02), N07BB and N07BC), obtained from the Danish National Prescription Register [22]. The choices of ATC codes were based on advice from a senior psychiatrist on medication most often used for treating mental disorder diagnoses in Denmark, with exclusion of medication that often is used to treat other types of disorders. Medication use is defined as prescription filled in the period between half a year before and half a year after the adolescents'15th birthday.

#### SES measures

Equalized family income is a measure of the disposable income weighted by the number of people in the family obtained from the Register of Family Income [23]. The equalized family income was categorized according to the OECD definition of low (20% lowest), middle (60%) and high (20% highest) income group [24]. The adolescents were grouped with adolescent born the same year. We used the mean of the equalized family income the year before the adolescents' 15th birthday, the year of the 15th birthday and the year after the 15th birthday. The mean of 3 years was used to reduce information bias related to negative income for one year, e.g. because of financial loss due to a poor investment year [25]. If information on less than 3 years income was available in the period, this information was used.

Parents' highest educational level is categorized according to the International Standard Classification of Education (ISCED) into educational level group of short (up to secondary school (ISCED: 0–2), middle (upper secondary school, vocational education or short-cycle tertiary education (ISCED: 3–5), and long (bachelor's degree or higher (ISCED: 6–8)) [26]. Data on parents' educational level—defined as the highest level completed by either parent—was obtained on the adolescents' 15th birthday obtained from Register of the Highest Completed Education [27]. The middle group was used as the reference category in both SES measures, as it represents the most typical group in the population and enables comparisons relative to the average adolescent [28].

#### Covariates

Covariates consist of sex, country of origin, parents living together, family's mental disorder, adolescents' multimorbidity, and family's multimorbidity. The adolescents' sex was defined as male or female according to their legal registered sex. The country of origin is coded as born in Denmark or born outside Denmark. Parents living together was defined as the legal parents living in the same household as the adolescent from birth until the time of the 15th birthday. The measure was dichotomized into parents living together since birth or parents not living together in the period since birth. Data on sex, origin, and parents were living together was obtained from the Population Register [29].

Family's mental disorder was defined as any mental disorder diagnosis since birth of the family member or from 1995 until the 15th year birthday of the adolescent or any prescription for psychopharmacological medication. Siblings were defined as children or adolescents (< 25 years old) living in the same household as the adolescents at the time of the 15th birthday. Parents was defined as legal parents. Mental disorder in siblings and parents was measured dichotomously, indicating whether or not mental disorder was present in the family. The medication use was measured half a year before and half a year after the 15th birthday. Multimorbidity of the adolescents, siblings and parents was defined by a modified version of the Nordic Multimorbidity Index (NMI). In the NMI, 50 predictors of multimorbidity are weighted from −2 to 22. The index date was defined as the 15th birthday of the adolescent and the predictors were based on ICD-10 codes in the period 5 years before the index date and ATC codes in the period 6 month before the index date. We excluded ICD-10 codes related to mental disorder diagnosis (F10 and F17) and ACT codes of psychopharmacological mediation (N05A, N05BA, N05CD, N05CF, N06A and N07BC), as they are part of the outcome for the adolescents, and part of the covariate of mental disorder in family for siblings and parents [30]. Data on health and medication was obtained from the National Patient Registry and Danish National Prescription Register [20, 22].

### Missing data

To account for missing data on income and educational level, multiple imputations with chained equations and 10 iterations are used. The models were built on information about year, sex, multimorbidity of the adolescents, the parents and the siblings, psychotropic medication use of the adolescent, any mental disorder diagnosis and diagnosis group of the adolescent, mental disorder in siblings and parents, parents living together, parents’ educational level, and family income 2.5 year before the 15th birthday and 2.5 year after.

### Statistical methods

#### Descriptive statistics

The characteristics of the adolescents in terms of mental disorder diagnoses, medication use, SES groups defined by income, SES groups defined by parents’ educational level, origin, parents living together, and NMI are presented in seven 3-year periods from 2002–2022. Mental disorder diagnoses and medication use are also presented sex-stratified and stratified by the SES measures (equalized family income and parents'highest educational level). For each mental disorder diagnosis, the mean age of onset is presented.

#### Main analyses

The odds ratios (OR) of the equalized family income and mental health measures, along with the 95% confidence intervals (CI), were estimated using logistic regression adjusted for country of origin, the adolescent's multimorbidity, siblings' multimorbidity, parents' multimorbidity, siblings' mental disorder, parents' mental disorder, and parents living together.

The ORs for the parents' highest educational level and mental health measures were estimated using logistic regression adjusted for country of origin, parents' chronic illness and parents' mental disorder. The selection of covariates for adjustment was based on existing literature and the construction of directed acyclic graphs (DAGs) (Supplementary Fig. 1 & 2).

#### Sensitivity analyses

The used thresholds for equalized family income were explored by expanding the time frame to the mean of 5 years instead of 3 years and use of tertiles to define low-, middle-, and high-income groups. Moreover, sensitivity analyses using only mental disorder diagnosis from age 7 instead of year 1995 or birth was calculated to explore the effect of the lack of data in the first years of life in the earliest cohorts.

The data were analysed on the secure server of Statistics Denmark. All analyses were conducted in Stata version 17 [31]. Plots and graphs were performed in R Studio (Version 4.4.1).

## Results

Descriptive results of the characteristics of the included adolescents grouped in 3-year periods showed slightly higher prevalences of males compared to females throughout the period 2002–2022 (Table 1). The prevalence of adolescents with any mental disorder diagnosis has increased in the past decades from 6% in 2002–2004 to 19% in 2020–2022 with the highest prevalences of behavioural disorders (7%) and developmental disorders (5%). Generally, a tendency of earlier mean age-of-onset for some mental disorder diagnoses was observed, while the age-of-onset for mood disorders, eating disorders, and behavioural disorders was relatively stable. Likewise, the prevalence of medication use increased in the same period from 2% in 2002–2004 to 9% in 2020–2022. A sensitivity analysis showed that the prevalence of ADHD medication use increased in the first two cohorts and then remained stable, accounting for approximately 50% of all psychotropic prescriptions from 2008 onwards (Supplementary Table 1). The adolescents’ parents have a longer education in the later years and there is an increasing prevalence of adolescents with origins outside of Denmark. Data were imputed on < 0.1% of household income and 0.6% of parental educational level. Missing data on parental education were more common among individuals with origins other than Denmark, likely due to incomplete registration of foreign educational histories (Supplementary Table 2). The prevalence of adolescents living with both parents have remained stable at around 57% throughout the period. The general multimorbidity of the adolescents increased from a mean on 0.19 (0.19–0.19) in 2002–2004 to 0.22 (0.22–0.23) in 2020–2022. As the adolescents were grouped in income-groups with adolescents born the same year according to the OECD definition, and afterwards the populations were grouped in 3-year-cohorts, the size of the income groups differs slightly from the 20/60/20 distribution.

**Table 1:**
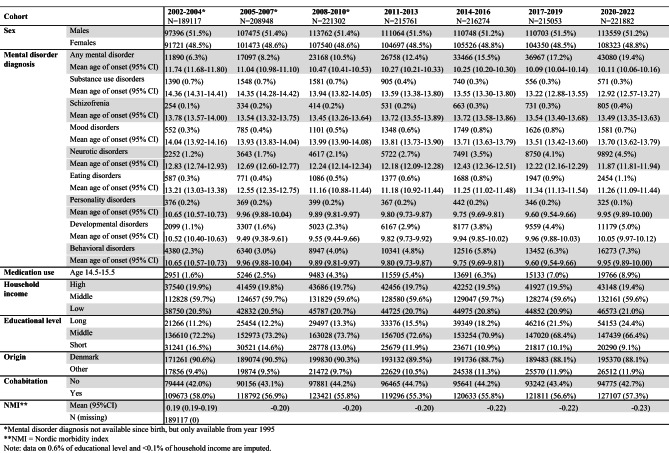
Characteristics of 15-year-olds grouped in 3-year periods

Sex specific analyses showed that males had a higher prevalence of any mental disorder and medication use than females in all seven periods. Generally, males had a higher prevalence of developmental disorders and behavioral disorder, while females had a slightly higher prevalence of mood disorders, eating disorders, and anxiety-related disorders (Supplementary Table 3).

### SES-specific prevalences

Income group specific analyses showed the highest prevalences of any mental disorder in the low-income group and the lowest prevalences in the high-income group across all time periods (Supplementary Table 4). Diagnosis-specific analyses showed the highest prevalences of substance use disorders, psychotic disorders, anxiety-related disorders, developmental disorders, and behavioral disorders in the low-income group and the lowest prevalences in the high-income group across all time periods. Likewise, the prevalences of medication use was highest in the low-income group and lowest in the high-income group across time periods. Educational level specific analyses showed that for most time periods, the short-education group had the highest prevalence of any mental disorder while the long-education group had the lowest prevalences (Supplementary Table 5). However, in the latest period, the short- and middle-education group had the same prevalences of any mental disorder diagnosis. The same tendency was present regarding medication use. The diagnosis specific analyses showed higher prevalences of behavioral disorders in the short-education group and lowest prevalences in the long-education group.

### Associations between SES measures and mental disorder diagnoses

**Fig. 1:**
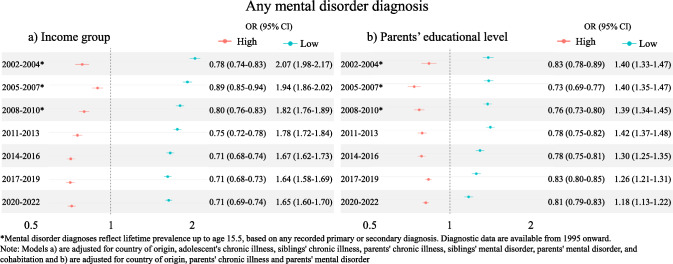
Odds ratios (OR) of any mental disorder diagnoses by (**a**) income group and (**b**) parents’ educational level

Analyses of odds for having any mental disorder diagnosis by income group showed that the high-income group consistently had lower odds compared to the middle-income group, while the low-income group consistently had higher odds compared to the middle-income group (Fig. 1a). A similar tendency was found when analyzing the odds for mental disorder diagnosis by educational level, where the odds were consistently lower for the long-education group compared to the middle-education group and consistently higher for the short-education group (Fig. 1b). The strengths of the associations between short-educational level and mental disorder diagnoses appeared to decrease over time, and a similar pattern was observed for low-income compared to the middle-income group. In contrast, the associations between long educational level and high income relative to the middle group remained stable over time.

**Fig. 2:**
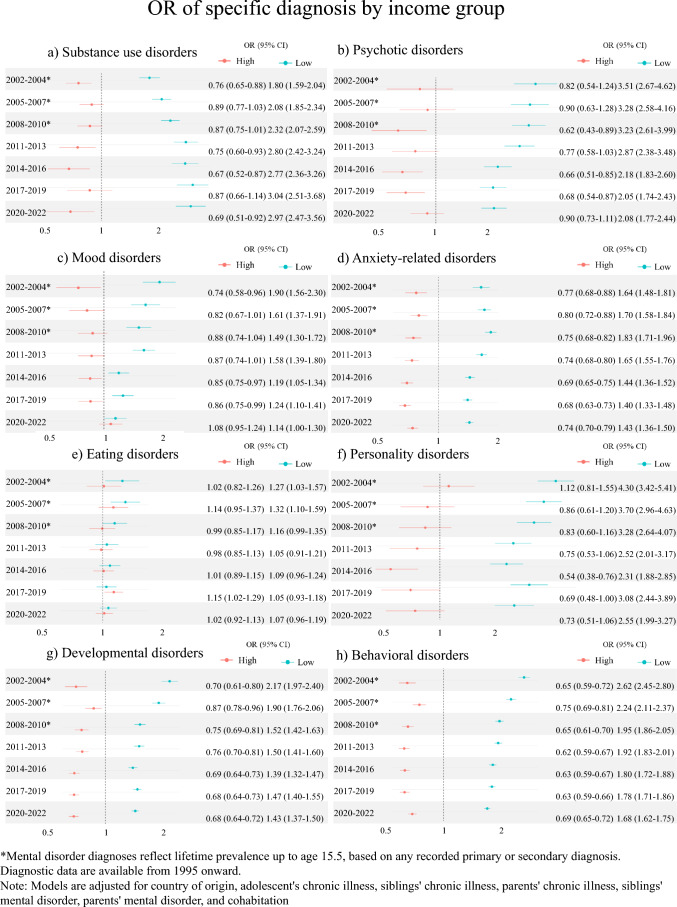
Odds Ratios (OR) of specific mental disorder diagnoses by income group for (**a**) Substance use disorders, (**b**) Psychotic disorders, (**c**) Mood disorders, (**d**) Anxiety-related disorders, (**e**) Eating disorders, (**f**) Personality disorders, (**g**) Developmental disorders, and (**h**) Behavioral disorders

Diagnosis specific analyses of income showed that the low-income group generally had higher odds compared to the middle-income group across diagnoses and across time periods, while the opposite was present for the high-income group compared with the middle-income group (Fig. 2A-H). The tendency was most pronounced for substance use disorder and personality disorder. For eating disorder, no association was found between income groups and odds of diagnoses, only for the low-income group compared with the middle-income group in the two earliest periods. A general pattern of decreasing associations between the low-income group and the specific mental disorder diagnosis was present in all diagnosis but substance use disorder, where the association seemed to strengthen over time, and anxiety-related disorder, where the association was stable over time.

**Fig. 3:**
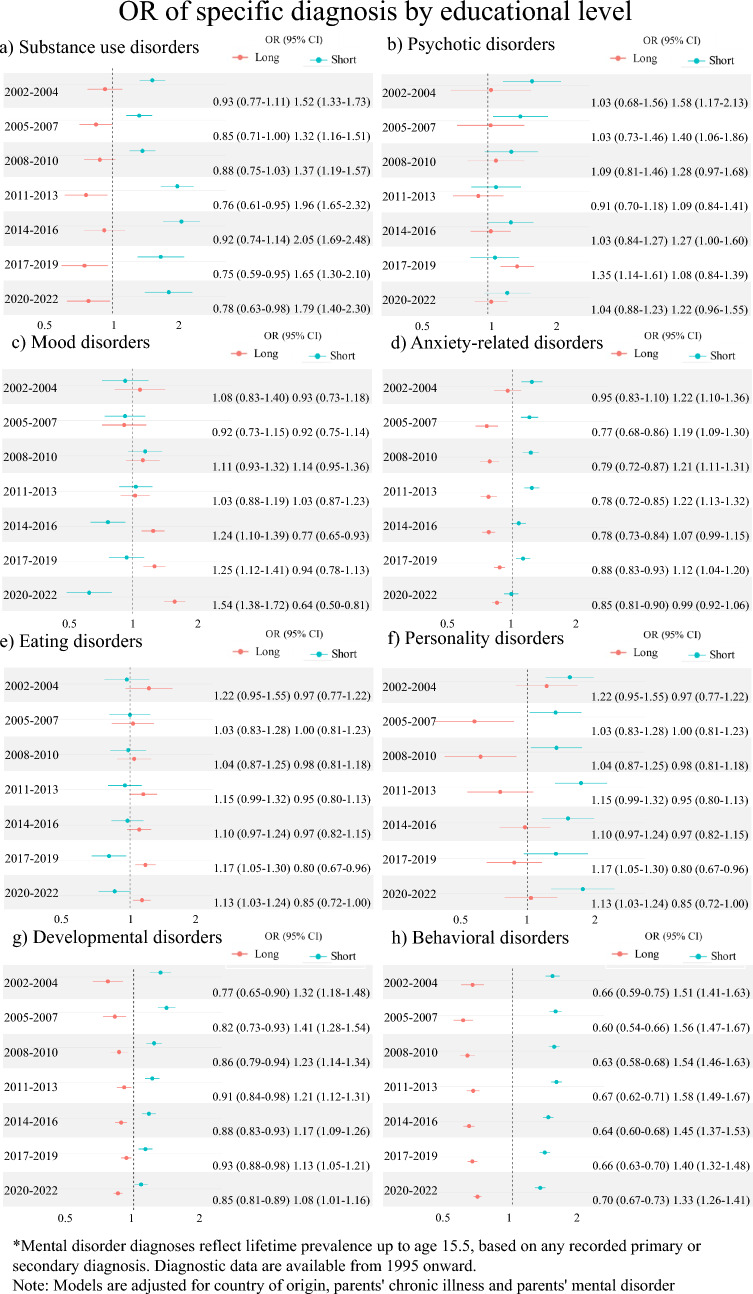
Odds ratios (OR) of specific mental disorder diagnoses by educational level for (**a**) Substance use disorders, (**b**) Psychotic disorders, (**c**) Mood disorders, (**d**) Anxiety-related disorders, (**e**) Eating disorders, (**f**) Personality disorders, (**g**) Developmental disorders, and (**h**) Behavioral disorders

Diagnosis-specific analyses of educational level showed that for most disorders, the odds were higher for the short-education group compared to the middle-education group, and lower for the long-education group compared to the middle-education group (Fig. 3A-H). For mood disorders and eating disorders the tendency was reversed with higher odds for the long-education group compared to the middle-education group and lower odds for the short-education group compared to the middle-education group. For developmental disorders, the associations were smaller in the recent years. Over time, the associations between educational level and anxiety-related-, developmental-, and behavioral disorder decreased, the associations between educational level and substance use-, mood-, and eating strengthened, and the associations between educational level and psychotic disorders and personality disorder were stable. The association between educational level and mood disorders reversed over time, so the long-education group had a higher odds than the middle-education group, while the low-education group had lower odds than the middle-education group.

### Associations between SES measures and medication use

**Fig. 4:**
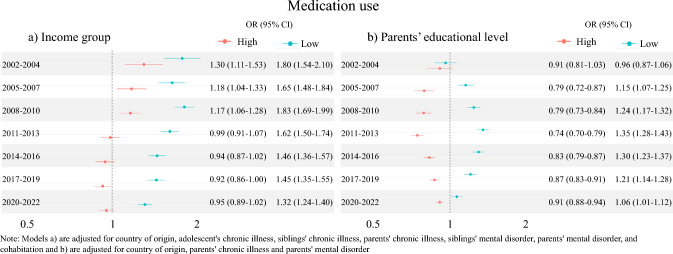
Odds Ratios (OR) of any medication use by (**a**) income group and (**b**) parents’ educational level

Analyses of medication use by income level showed higher odds of medication use for the high-income group compared to the middle-income group in the three periods from 2002–2010 and comparable odds in the remaining four periods from 2011–2022 (Fig. 4A). The low-income group had consistently higher odds for medication use compared with the middle-income group. 

Analyses of medication use by educational level showed lower odds of medication use for the high-education group compared to the middle-education group and higher odds of medication use for the low-education group compared to the middle-education group in the periods from 2005–2022 (Fig. 4B). In the period 2002–2004, the odds were comparable between groups.

### Sensitivity analyses

Sensitivity analyses of alternative measures of income (5-year-mean instead of 3 and grouped as tertiles instead of the OECD classification) did not show notably different results than the main analyses. Neither did the sensitivity analyses of mental disorder diagnosis measured from age 7 change the associations, even though the absolute estimates showed higher prevalence and earlier age-of-onset of mental disorder in the more recent cohorts (Supplementary Fig. 3 & Table 4).

## Discussion

### Key results

This study found that the prevalence of mental disorder diagnoses and medication use among 15-year-old adolescents increased during the past decades, with developmental disorder and behavioural disorder being most frequent and showing the largest increases. Higher prevalences of mental disorder diagnoses and medication use were consistently observed in the low-income and short-education groups. However, over time, the prevalences of mental disorder diagnoses and medication use between educational groups and income-groups became more similar. The associations between short-educational level and mental disorder diagnoses decreased over the study period, while the associations between short-educational level and medication use increased until 2011–2013 and then decreased. In contrast, associations between long-educational level and mental disorder diagnoses remained stable over time, while associations with medication use first increased until 2011–2013 and then decreased. Time trends varied across specific mental disorder diagnosis: for income, a general decrease in associations with most mental disorder diagnosis and low-income was observed, except for substance use disorder, where the associations seemed to increase, and anxiety-related disorder, where the association remained stable. For shot-educational level, associations with anxiety-related-, developmental-, and behavioral disorder decreased over time, while associations with substance use-, mood-, and eating disorders increased over time. Associations with psychotic- and personality disorder remained stable. Notably, associations between educational level and mood disorder were reversed compared to other disorders: the long-education group had higher odds than the middle-education group, and the low-education group having lower odds than the middle-education group. Regarding general time trends in income, low-income had decreasing associations with both mental disorder diagnosis and medication use over time, while associations between high-income and mental disorder diagnoses remained stable.

### Discussion of results

We observed an increasing prevalence of mental disorder diagnoses and medication use among adolescents over the study period, aligning with previous research [3, 7, 8, 32, 33]. Importantly, a clear tendency of social inequality was present with adolescents from the low-income and short-education groups exhibiting higher odds of being diagnosed with mental disorders and of medication use compared to their peers from middle-income and middle-education groups, underscoring their heightened vulnerability. These findings align with previous research on the association between SES and mental health [11–17]. To our knowledge, this study is the first to show a trend of less social inequality over time with decreasing associations between low-SES and mental health.

Interestingly, a tendency of decreasing associations between the low-income group compared to the middle-income group and mental health was found, even though the income inequality has increased the past decades, reflected in the GINI coefficient [9]. In our data, we saw a tendency of rising inequality between income groups in the later cohorts reflected in a higher relative mean income in the high-income group compared to the mean income in middle-income group, and a lower relative mean income in the low-income group compared to the mean income in the middle-income group. Therefore, the decreasing associations cannot be explained by the definition of income-groups over time. However, the decreasing associations of short-educational level compared to middle and mental health over time may be explained by changes in educational level. A shift has been recognized in the composition of educational groups, as more individuals pursue higher education, thereby making the long-education group more heterogeneous while shrinking the short-education group, resulting in people from the middle and short group becoming more alike.

The observed differences in associations between income and education with mental health over time may reflect the distinct dimensions of social status they capture—income reflects economic resources, while parental education reflects cognitive skills and cultural capital—each potentially influencing mental health in different ways.

The increasing prevalence of poor mental health has fuelled the ongoing debate about its underlying causes which may also explain the lower inequality. Key hypotheses include improved diagnostic practices, a lowered threshold for diagnosis, increased psychologization, or a genuine increase in mental health problems [34–36]. A prominent area of discussion focuses on potential over-diagnosis, particularly among young people with ADHD. For instance, Australian research suggests that the substantial increase in ADHD diagnoses was not accompanied by a corresponding increase in symptoms such as hyperactivity and inattention [36]. Psychologization refers to the growing tendency to interpret challenges through a psychological lens, often framing difficulties in terms of mental health diagnoses. This phenomenon may reflect broader societal shifts, where young people increasingly rely on psychological explanations for their struggles [34, 35]. Over-diagnosis and psychologization may particularly affect individuals from high SES, who are more likely to engage in public debates and be influenced by discussions around mental health. They are also better equipped to navigate the healthcare system and consistently seek treatment for their children. As a result, they may be more susceptible to over-diagnosis and the psychotropic medications that often follow [37, 38]. This may help explain the decreasing associations between both income and educational level and poor mental health.

The latter hypothesis, of a genuine increase in adolescents’ mental health problems, suggests that adolescents may be experiencing increasing performance pressure from academic, social, and cultural expectations, which may act as a mechanism driving the observed increase in mental health problems [34, 39]. Qualitative studies offer insights into social inequality in mental health, suggesting that adolescents from low-SES backgrounds face compounded challenges. The dual burden of performance pressure and financial strain makes adolescents more susceptible to adverse outcomes when encountering difficulties. Conversely, a new trend has emerged: adolescents from middle- and high-SES backgrounds are increasingly susceptible to mental health problems due to rising academic, social, and cultural expectations [34]. Our results of decreased association between SES and poor mental health may reflect a societal shift, indicating that increased pressure on adolescents is now affecting both high- and middle-SES groups as well.

Diagnosis-specific analyses showed associations between the low-SES groups and several diagnoses compared to the middle-SES groups. Moreover, the associations changed over time, especially the associations between educational level and specific mental diagnoses showed diverse patterns over time. The associations between substance use disorders and both educational level and income strengthened over time. Substance use disorders are known to have heritable components [40]. Although our analyses adjusted for parental mental health diagnoses and psychotropic medication use, residual confounding may persist due to undiagnosed conditions. Undiagnosed conditions might especially be prevalent in parents because of a birth cohort effect of mental disorder diagnoses. Momen et al. have demonstrated that the age of onset for several mental disorder diagnoses has shifted downward in the past years [7]. Therefore, parents may be more prone to be undiagnosed. Undiagnosed mental health conditions in parents could affect labor market attachment and subsequently household income and the parents’ educational level, and thereby explain the results. In contrast, for mood disorders and eating disorders, the associations changed over time to higher odds for long-education compared to the middle-education group while the odds were lower for the short-education compared to the middle-education group in the later cohorts. Likewise, the associations of income and mood- and eating disorders decreased over time and were comparable across groups in the later cohorts. This pattern suggests a different dynamic than for other diagnoses, that might reflect a higher likelihood among high SES parents to navigate the healthcare system and consistently seek treatment for their children within these specific diagnoses [37, 38]. Moreover, our diagnosis-specific analyses showed that the prevalence of developmental disorders was more equally distributed across educational levels in the latest cohort, especially in 2020–2022, where the middle-education group had the highest prevalence. Diagnoses of developmental disorders might drive some of the general decreased associations between educational level and mental disorder diagnoses over time.

Finally, the associations between SES and medication use decreased over the years, which partly could be explained by a change in prescription practices since 2014. Since the change in prescription practices, general practitioners (GP) were no longer allowed to prescribe antidepressants drugs for people under 25 years, only psychiatrist were allowed to prescribe this kind of medication [6]. Nevertheless, the overall prevalence of medication use continued to increase, which means that psychiatrists are prescribing more. This may be partly explained by the fact that approximately half of all prescriptions were for ADHD medication, as shown in the supplementary analyses. The decreased social inequality in medication use could be explained by the low-income group and short-education group being more affected by the change in prescription practices resulting in not getting as many prescriptions at the psychiatrist as they got at the GPs. It is also relevant to consider if other mental health treatments have shifted from GPs to hospital-based settings, which could affect the interpretation of changes observed over time.

### Strengths

Our study has several strengths. First, the use of national register data ensured comprehensive coverage of all Danish adolescents, minimizing the risk of selection bias. Second, missing data were minimal, and the application of multiple imputation methods combined with strong auxiliary variables ensured robust handling of any missingness. These features enhance the reliability and generalizability of our findings. Third, the rich information from registers and the possibility to connect with information from families makes it possible to adjust for several relevant confounders of the associations between SES and mental health measures.

### Limitations

Despite its strengths, the study has several limitations. First, the data break with no information on outpatients’ mental disorder diagnoses before 1995 makes the comparison of cohorts over time difficult for the periods 2002–2009 since no information is present in the first years of the adolescents’ life in these cohorts. The results on prevalences of mental disorder diagnoses from the first three cohorts should be interpreted with caution as mental disorder diagnoses might be underreported. However, the sensitivity analyses using diagnosis from age 7 for all cohorts did not alter the conclusions of the relative measures and therefore, the results on the associations are considered valid. Second, our analyses were constrained by the variables available in the registers, leaving the possibility of residual confounding due to unmeasured factors such as parenting style, community characteristics, or individual coping mechanisms [41–43]. Third, the reliance on register data means that only severe mental disorders requiring hospital-based care (including outpatient visits) were captured. This limitation may lead to an underrepresentation of cases among adolescents from low-income and short education groups, as more resourceful families may be more proactive in navigating the healthcare system [37, 38]. However, adolescents from high-income and long-education groups might have more resources to go to private psychiatrists which could lead to underrepresentation of cases among adolescents from high-income and long-education groups. Moreover, this study is likely only to capture the most severe cases of poor mental health, as it relies on mental disorder diagnoses and psychotropic medication use from national registers. Weye et al. found that only 15% of individuals with current depression were captured in the hospital registers, while 51% were identified through prescription [44]. Therefore, a substantial underrepresentation of less severe cases is likely. Finally, because SES and mental health were measured simultaneously, our findings cannot establish causal relationships. Thus, our results could indicate that mental health of the adolescents during their upbringing could affect the parents’ ability to obtain a higher education and/or their ability to obtain a high-income. Conversely, low-SES of the parents could reflect low resources in the family which can lead to poor mental health of the adolescents.

## Conclusion

This study highlights persistent but evolving social inequalities in adolescent mental health in Denmark from 2002 to 2022. While the prevalence of adolescents’ mental health diagnoses and medication use increased over the period, a general trend towards decreasing OR between low- and middle-SES groups were observed, while the association between high- and middle-SES groups remained relatively stable. However, changes in inequality patterns varied by diagnosis, with some conditions showing decreasing associations with SES and others showing increasing trends. These diagnosis-specific shifts suggest that broader societal changes may influence different mental disorder diagnoses in diverse ways. Future research should explore the mechanisms driving these diagnosis-specific trends and examine strategies to address social inequalities in adolescent mental health.

## Supplementary Information

Below is the link to the electronic supplementary material.

Supplementary material 1 (DOCX 628.7 kb)

## Data Availability

Data have been made available for the first author specifically via Statistics Denmark and can therefore not be shared. The codes are available from the corresponding author on reasonable request.
