# Supplementary material for: Social inequality in mental disorder diagnoses and psychotropic medication use among 15-year-old adolescents in Denmark from 2002–2022
Source: Soc Psychiatry Psychiatr Epidemiol. 2025 Jul 2;61(1):15–27. doi: 10.1007/s00127-025-02943-y (PMC12855378; doi:10.1007/s00127-025-02943-y)
Supplement: Supplementary file 1 — Supplementary material 1 (DOCX 628.7 kb) [file 127_2025_2943_MOESM1_ESM.docx]

# Supplementary

*Reading instruction Supplementary Figure 1 & 2*

A DAG is a Directed (implies direction) Acyclic (no cycles: a variable can't cause itself) Graph. DAGs are used to visualize the association between variables and help identify how to analyse an unbiased association between an

exposure and an outcome. Ancestors of both exposures and outcome must be adjusted for in an analysis to ensure no biased paths. In this study, the exposure and outcome happen at the same time. Therefore, the associations between outcome and exposure in the DAG's should be interpreted as potentially bidirectional.

| Main variables | Ancestors | Paths |
| --- | --- | --- |
| 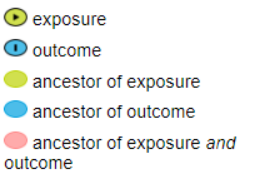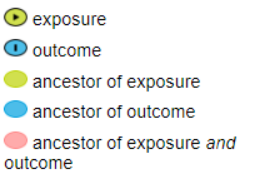Outcome  Exposure | 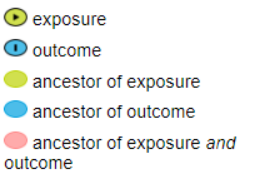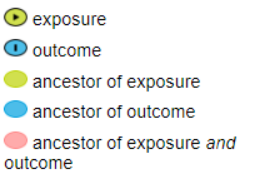Ancestor of exposure  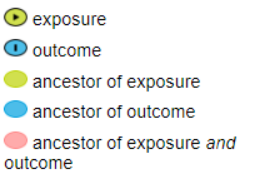Ancestor of outcome  Ancestor of both exposure and outcome | 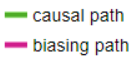Causal path  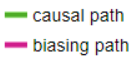Biased path |

**Supplementary Figure 1**: Equivalized family income & adolescent mental health

*
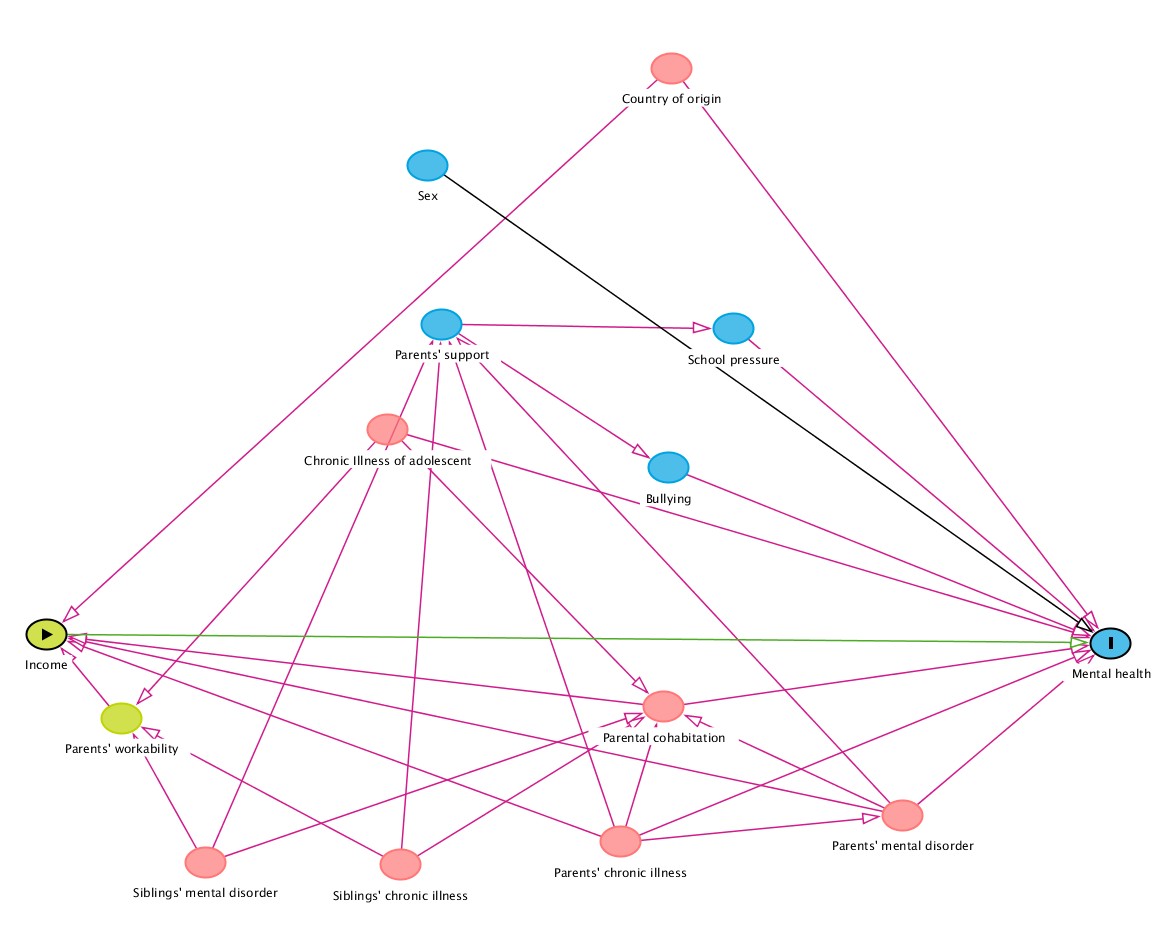
*

The model will be adjusted for; country of origin, adolescent's chronic illness, siblings' chronic illness, parents' chronic illness, siblings' mental disorder, parents' mental disorder and parental cohabitation.

**Supplementary Figure 2**: Parents' education & adolescent mental health *
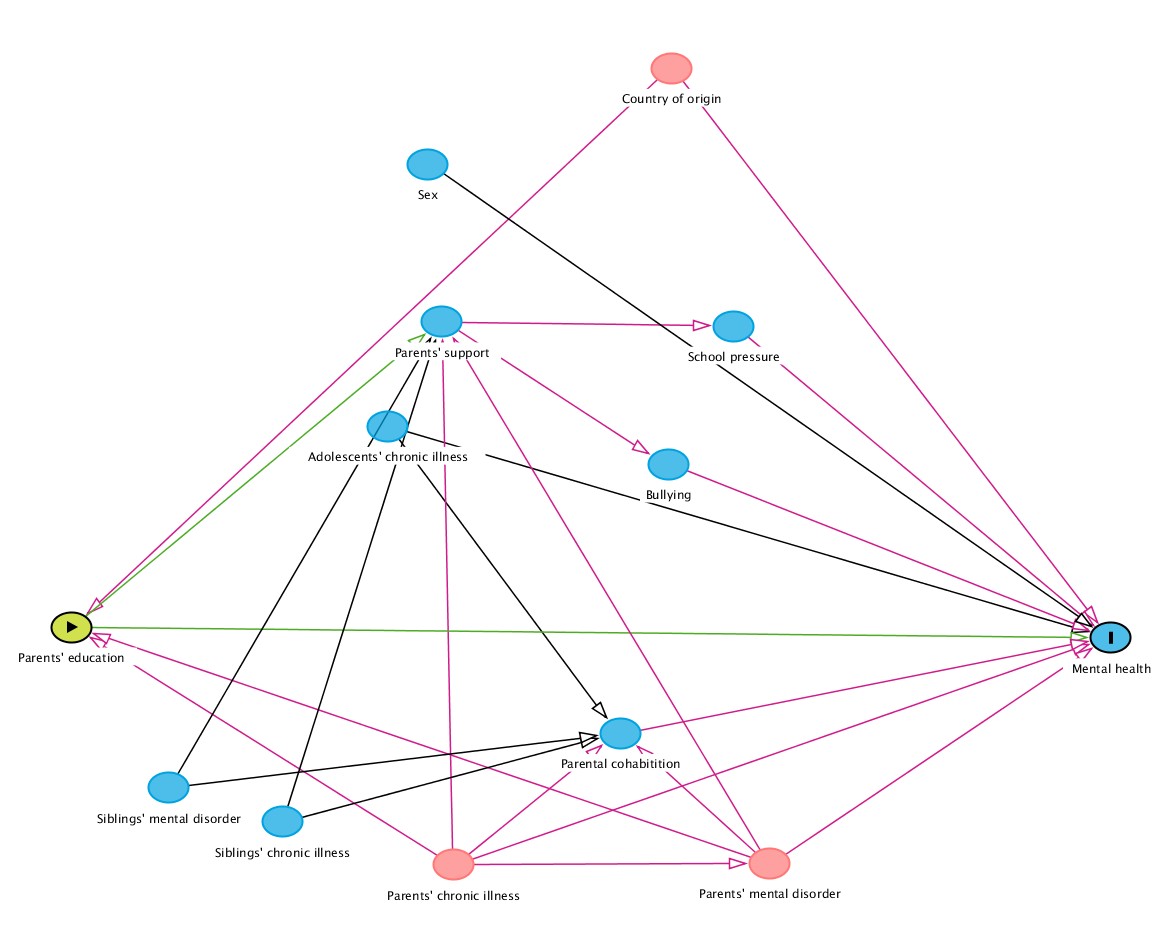
*

The model will be adjusted for; country of origin, parents' chronic illness and parents' mental disorder.

| Supplementary Table 1: Prevalence of ADHD medication use among all prescriptions for 15-year-olds, grouped in 3-year periods | | | | | | | |
| --- | --- | --- | --- | --- | --- | --- | --- |
| n(%) | **2002-2004** | **2005-2007** | **2008-2010** | **2011-2013** | **2014-2016** | **2017-2019** | **2020-2022** |
| Other psychotropic medication use | 1,662 (76%) | 2,449 (64%) | 3,272 (48%) | 3,858 (45%) | 4,423 (47%) | 4,888 (48%) | 7,042 (51%) |
| ADHD related medication use | 515 (24%) | 1,390 (36%) | 3,636 (53%) | 4,670 (55%) | 5,040 (53%) | 5,319 (52%) | 6,744 (49%) |
| Total prescriptions | 2,177 | 3,839 | 6,908 | 8,528 | 9,463 | 10,207 | 13,786 |

*Note: Individuals may appear more than once if they received both ADHD and other psychotropic medications.

| Supplementary Table 2: Parental educational level and missingness by country of origin | | | | | |
| --- | --- | --- | --- | --- | --- |
| N(%) | **Parental educational level** | | | | |
| Country of origin | **Long** | **Middle** | **Short** | **Missing** | **Total** |
| Denmark | 232,196 (17.5%) | 974,346 (73.3%) | 122,198 (9.2%) | 1,146 (0.1%) | 1,329,886 (100%) |
| Other | 16,199 (10.2%) | 77,233 (48.7%) | 56,690 (35.8%) | 8,329 (5.3%) | 158,451 (100%) |

| **Supplementary Table 3: Mental health measures of 15-year-olds grouped in 3-year periods stratified by sex** | | | | | | | | | | | | |
| --- | --- | --- | --- | --- | --- | --- | --- | --- | --- | --- | --- | --- |
| **Cohort** | | **Sex** | **Mental disorder diagnosis** | | | | | | | | | **Medication use** |
|  |  |  | Any mental disorder | Substance use disorders | Psychotic disorders | Mood disorders | Anxiety-related disorders | Eating disorders | Personality disorders | Developmental disorders | Behavioral disorders | Age 14.5-15.5 |
| **2002-2004*** | Males (N=97,396) | | 6792 (7.0%) | 703 (0.7%) | 116 (0.1%) | 193 (0.2%) | 863 (0.9%) | 119 (0.1%) | 107 (0.1%) | 1629 (1.7%) | 3062 (3.1%) | 1547 (1.6%) |
|  | Females (N=107,475) | | 5098 (5.6%) | 687 (0.7%) | 138 (0.2%) | 359 (0.4%) | 1389 (1.5%) | 468 (0.5%) | 269 (0.3%) | 470 (0.5%) | 1318 (1.4%) | 1404 (1.5%) |
| **2005-2007*** | Males (N=107,475) | | 9680 (9.0%) | 760 (0.7%) | 137 (0.1%) | 260 (0.2%) | 1358 (1.3%) | 177 (0.2%) | 95 (0.1%) | 2551 (2.4%) | 4342 (4.0%) | 2797 (2.6%) |
|  | Females (N=101,473) | | 7417 (7.3%) | 788 (0.8%) | 197 (0.2%) | 525 (0.5%) | 2285 (2.3%) | 594 (0.6%) | 274 (0.3%) | 756 (0.7%) | 1998 (2.0%) | 2449 (2.4%) |
| **2008-2010*** | Males (N=113,762) | | 13520 (11.9%) | 781 (0.7%) | 182 (0.2%) | 372 (0.3%) | 1811 (1.6%) | 294 (0.3%) | 107 (0.1%) | 3846 (3.4%) | 6127 (5.4%) | 5561 (4.9%) |
|  | Females (N=107,540) | | 9648 (9.0%) | 800 (0.7%) | 232 (0.2%) | 729 (0.7%) | 2806 (2.6%) | 792 (0.7%) | 292 (0.3%) | 1177 (1.1%) | 2820 (2.6%) | 3922 (3.6%) |
| **2011-2013** | Males (N=111,064) | | 15561 (14.0%) | 781 (0.7%) | 218 (0.2%) | 457 (0.4%) | 2255 (2.0%) | 310 (0.3%) | 83 (0.1%) | 4655 (4.2%) | 7129 (6.4%) | 6965 (6.3%) |
|  | Females (N=104,697) | | 11197 (10.7%) | 800 (0.7%) | 313 (0.3%) | 891 (0.9%) | 3467 (3.3%) | 1067 (1.0%) | 284 (0.3%) | 1512 (1.4%) | 3212 (3.1%) | 4594 (4.4%) |
| **2014-2016** | Males (N=110,748) | | 18825 (17.0%) | 454 (0.4%) | 255 (0.2%) | 564 (0.5%) | 2944 (2.7%) | 438 (0.4%) | 116 (0.1%) | 5816 (5.3%) | 8330 (7.5%) | 7995 (7.2%) |
|  | Females (N=105,526) | | 14641 (13.9%) | 451 (0.4%) | 408 (0.4%) | 1185 (1.1%) | 4547 (4.3%) | 1250 (1.2%) | 326 (0.3%) | 2361 (2.2%) | 4186 (4.0%) | 5696 (5.4%) |
| **2017-2019** | Males (N=110,703) | | 20979 (19.0%) | 362 (0.3%) | 269 (0.2%) | 556 (0.5%) | 3638 (3.3%) | 576 (0.5%) | 73 (0.1%) | 6625 (6.0%) | 8943 (8.1%) | 8786 (7.9%) |
|  | Females (N=104,350) | | 15988 (15.3%) | 378 (0.4%) | 462 (0.4%) | 1070 (1.0%) | 5112 (4.9%) | 1371 (1.3%) | 273 (0.3%) | 2934 (2.8%) | 4509 (4.3%) | 6347 (6.1%) |
| **2020-2022** | Males (N=113,559) | | 23367 (20.6%) | 299 (0.3%) | 268 (0.2%) | 467 (0.4%) | 4013 (3.5%) | 795 (0.7%) | 47 (0.0%) | 7310 (6.4%) | 10194 (9.0%) | 10596 (9.3%) |
|  | Females (N=108,323) | | 19713 (18.2%) | 257 (0.2%) | 537 (0.5%) | 1114 (1.0%) | 5879 (5.4%) | 1659 (1.5%) | 278 (0.3%) | 3869 (3.6%) | 6079 (5.6%) | 9170 (8.5%) |

*Mental disorder diagnoses reflect lifetime prevalence up to age 15.5, based on any recorded primary or secondary diagnosis. Diagnostic data are available from 1995 onward.

| **Supplementary Table 4: Mental health measures of 15-year-olds grouped in 3-year periods stratified by income group** | | | | | | | | | | | |
| --- | --- | --- | --- | --- | --- | --- | --- | --- | --- | --- | --- |
| **Cohort** | **Income group** | **Mental disorder diagnosis** | | | | | | | | | **Medication use** |
|  |  | Any mental disorder | Substance use disorders | Psychotic disorders | Mood disorders | Anxiety-related disorders | Eating disorders | Personality disorders | Developmental disorders | Behavioral disorders | Age 14.5-15.5 |
| **2002-2004*** | High (N=37,540**)** | 7577 (4.0%) | 987 (0.5%) | 141 (0.1%) | 368 (0.2%) | 1562 (0.8%) | 569 (0.3%) | 252 (0.1%) | 1335 (0.7%) | 2363 (1.2%) | 2504 (1.3%) |
|  | Middle (N=112,828) | 10407 (5.5%) | 1296 (0.7%) | 176 (0.1%) | 503 (0.3%) | 2083 (1.1%) | 565 (0.3%) | 230 (0.1%) | 1887 (1.0%) | 3667 (1.9%) | 2561 (1.4%) |
|  | Low (N=38,749) | 20386 (10.8%) | 2055 (1.1%) | 591 (0.3%) | 874 (0.5%) | 3411 (1.8%) | 669 (0.4%) | 922 (0.5%) | 3455 (1.8%) | 8409 (4.4%) | 4519 (2.4%) |
| **2005-2007*** | High (N=41,459) | 12116 (5.8%) | 1169 (0.6%) | 202 (0.1%) | 580 (0.3%) | 2550 (1.2%) | 811 (0.4%) | 217 (0.1%) | 2550 (1.2%) | 4037 (1.9%) | 3845 (1.8%) |
|  | Middle (N=124,657) | 15094 (7.2%) | 1333 (0.6%) | 235 (0.1%) | 734 (0.4%) | 3341 (1.6%) | 719 (0.3%) | 258 (0.1%) | 2975 (1.4%) | 5500 (2.6%) | 4620 (2.2%) |
|  | Low (N=42,832) | 27748 (13.3%) | 2542 (1.2%) | 751 (0.4%) | 1132 (0.5%) | 5581 (2.7%) | 883 (0.4%) | 839 (0.4%) | 5005 (2.4%) | 11015 (5.3%) | 8425 (4.0%) |
| **2008-2010*** | High (N=43,686) | 15066 (6.8%) | 1089 (0.5%) | 182 (0.1%) | 876 (0.4%) | 2933 (1.3%) | 1018 (0.5%) | 228 (0.1%) | 3571 (1.6%) | 5167 (2.3%) | 6818 (3.1%) |
|  | Middle (N=131,829) | 21160 (9.6%) | 1298 (0.6%) | 304 (0.1%) | 1042 (0.5%) | 4150 (1.9%) | 1059 (0.5%) | 285 (0.1%) | 4867 (2.2%) | 8155 (3.7%) | 8471 (3.8%) |
|  | Low (N=45,787) | 36680 (16.6%) | 2866 (1.3%) | 952 (0.4%) | 1484 (0.7%) | 7569 (3.4%) | 1228 (0.6%) | 889 (0.4%) | 6858 (3.1%) | 14833 (6.7%) | 14940 (6.8%) |
| **2011-2013** | High (N=42,456) | 17167 (8.0%) | 503 (0.2%) | 285 (0.1%) | 1047 (0.5%) | 3695 (1.7%) | 1296 (0.6%) | 208 (0.1%) | 4401 (2.0%) | 5732 (2.7%) | 7923 (3.7%) |
|  | Middle (N=128,580) | 24998 (11.6%) | 703 (0.3%) | 398 (0.2%) | 1269 (0.6%) | 5368 (2.5%) | 1379 (0.6%) | 297 (0.1%) | 6002 (2.8%) | 9582 (4.4%) | 10723 (5.0%) |
|  | Low (N=44,725) | 40923 (19.0%) | 1867 (0.9%) | 1148 (0.5%) | 1862 (0.9%) | 8664 (4.0%) | 1447 (0.7%) | 719 (0.3%) | 8317 (3.9%) | 16899 (7.8%) | 17415 (8.1%) |
| **2014-2016** | High (N=42,252) | 21457 (9.9%) | 369 (0.2%) | 358 (0.2%) | 1428 (0.7%) | 4847 (2.2%) | 1623 (0.8%) | 195 (0.1%) | 5467 (2.5%) | 7171 (3.3%) | 9014 (4.2%) |
|  | Middle (N=129,047) | 32645 (15.1%) | 583 (0.3%) | 577 (0.3%) | 1785 (0.8%) | 7450 (3.4%) | 1674 (0.8%) | 387 (0.2%) | 8251 (3.8%) | 11939 (5.5%) | 13310 (6.2%) |
|  | Low (N=44,975) | 47102 (21.8%) | 1539 (0.7%) | 1197 (0.6%) | 1948 (0.9%) | 10094 (4.7%) | 1789 (0.8%) | 832 (0.4%) | 10512 (4.9%) | 19192 (8.9%) | 19177 (8.9%) |
| **2017-2019** | High (N=41,927) | 24046 (11.2%) | 339 (0.2%) | 415 (0.2%) | 1303 (0.6%) | 5642 (2.6%) | 2113 (1.0%) | 174 (0.1%) | 6360 (3.0%) | 7699 (3.6%) | 9776 (4.5%) |
|  | Middle (N=128,274) | 36167 (16.8%) | 411 (0.2%) | 645 (0.3%) | 1633 (0.8%) | 8823 (4.1%) | 1913 (0.9%) | 268 (0.1%) | 9575 (4.5%) | 12899 (6.0%) | 14462 (6.7%) |
|  | Low (N=44,852) | 51332 (23.9%) | 1175 (0.5%) | 1271 (0.6%) | 1908 (0.9%) | 11445 (5.3%) | 1889 (0.9%) | 729 (0.3%) | 12505 (5.8%) | 20411 (9.5%) | 22061 (10.3%) |
| **2020-2022** | High (N=43,148) | 29183 (13.2%) | 283 (0.1%) | 576 (0.3%) | 1522 (0.7%) | 6690 (3.0%) | 2412 (1.1%) | 180 (0.1%) | 7333 (3.3%) | 10187 (4.6%) | 14157 (6.4%) |
|  | Middle (N=132,161) | 42079 (19.0%) | 440 (0.2%) | 685 (0.3%) | 1551 (0.7%) | 9739 (4.4%) | 2468 (1.1%) | 260 (0.1%) | 11257 (5.1%) | 15679 (7.1%) | 19136 (8.6%) |
|  | Low (N=46,573) | 58795 (26.5%) | 1210 (0.5%) | 1358 (0.6%) | 1720 (0.8%) | 13292 (6.0%) | 2454 (1.1%) | 643 (0.3%) | 14521 (6.5%) | 23597 (10.6%) | 26751 (12.1%) |

* Mental disorder diagnoses reflect lifetime prevalence up to age 15.5, based on any recorded primary or secondary diagnosis. Diagnostic data are available from 1995 onward.

| **Supplementary Table 5: Mental health measures of 15-year-olds grouped in 3-year periods stratified by educational level** | | | | | | | | | | | |
| --- | --- | --- | --- | --- | --- | --- | --- | --- | --- | --- | --- |
| **Cohort** | **Educational level** | **Mental disorder diagnosis** | | | | | | | | | **Medication use** |
|  |  | Any mental disorder | Substance use disorders | SchizophreniPsychotic disorders | Mood disorders | Anxiety-related disorders | Eating disorders | Personality disorders | Developmental disorders | Behavioral disorders | Age 14.5-15.5 |
| **2002-2004*** | Long (N=21,253**)** | 9464 (5.0%) | 1202 (0.6%) | 232 (0.1%) | 589 (0.3%) | 2028 (1.1%) | 695 (0.4%) | 400 (0.2%) | 1568 (0.8%) | 2751 (1.5%) | 2685 (1.4%) |
|  | Middle (N=136,626) | 11450 (6.1%) | 1307 (0.7%) | 228 (0.1%) | 556 (0.3%) | 2168 (1.1%) | 579 (0.3%) | 334 (0.2%) | 2074 (1.1%) | 4204 (2.2%) | 3009 (1.6%) |
|  | Short (N=31,238) | 15465 (8.2%) | 1883 (1.0%) | 382 (0.2%) | 510 (0.3%) | 2772 (1.5%) | 547 (0.3%) | 543 (0.3%) | 2570 (1.4%) | 6259 (3.3%) | 2878 (1.5%) |
| **2005-2007*** | Long (N=25,441) | 12087 (5.8%) | 1244 (0.6%) | 304 (0.1%) | 699 (0.3%) | 2665 (1.3%) | 781 (0.4%) | 206 (0.1%) | 2575 (1.2%) | 3613 (1.7%) | 3994 (1.9%) |
|  | Middle (N=152,954) | 16881 (8.1%) | 1517 (0.7%) | 314 (0.2%) | 808 (0.4%) | 3648 (1.7%) | 776 (0.4%) | 374 (0.2%) | 3239 (1.6%) | 6205 (3.0%) | 5309 (2.5%) |
|  | Short (N=30,553) | 22360 (10.7%) | 1957 (0.9%) | 461 (0.2%) | 743 (0.4%) | 4431 (2.1%) | 739 (0.4%) | 478 (0.2%) | 4258 (2.0%) | 9292 (4.4%) | 5976 (2.9%) |
| **2008-2010*** | Long (N=29,514) | 17128 (7.7%) | 1285 (0.6%) | 404 (0.2%) | 1139 (0.5%) | 3443 (1.6%) | 1097 (0.5%) | 233 (0.1%) | 4171 (1.9%) | 5356 (2.4%) | 7157 (3.2%) |
|  | Middle (N=163,026) | 22931 (10.4%) | 1535 (0.7%) | 392 (0.2%) | 1077 (0.5%) | 4613 (2.1%) | 1086 (0.5%) | 400 (0.2%) | 5011 (2.3%) | 8816 (4.0%) | 9538 (4.3%) |
|  | Short (N=28,762) | 30701 (13.9%) | 2144 (1.0%) | 548 (0.2%) | 1197 (0.5%) | 5842 (2.6%) | 1075 (0.5%) | 561 (0.3%) | 5965 (2.7%) | 13369 (6.0%) | 11555 (5.2%) |
| **2011-2013** | Long (N=33,377) | 20225 (9.4%) | 606 (0.3%) | 439 (0.2%) | 1312 (0.6%) | 4235 (2.0%) | 1500 (0.7%) | 246 (0.1%) | 5356 (2.5%) | 6531 (3.0%) | 8235 (3.8%) |
|  | Middle (N=156,731) | 26689 (12.4%) | 842 (0.4%) | 531 (0.2%) | 1356 (0.6%) | 5794 (2.7%) | 1364 (0.6%) | 351 (0.2%) | 6171 (2.9%) | 10280 (4.8%) | 11718 (5.4%) |
|  | Short (N=25,653) | 35669 (16.5%) | 1675 (0.8%) | 649 (0.3%) | 1349 (0.6%) | 7217 (3.3%) | 1296 (0.6%) | 619 (0.3%) | 7199 (3.3%) | 15666 (7.3%) | 14911 (6.9%) |
| **2014-2016** | Long (N=39,329) | 25844 (11.9%) | 573 (0.3%) | 617 (0.3%) | 2015 (0.9%) | 5707 (2.6%) | 1772 (0.8%) | 380 (0.2%) | 6942 (3.2%) | 7837 (3.6%) | 10707 (5.0%) |
|  | Middle (N=153,268) | 34341 (15.9%) | 679 (0.3%) | 646 (0.3%) | 1756 (0.8%) | 7852 (3.6%) | 1681 (0.8%) | 427 (0.2%) | 8339 (3.9%) | 12960 (6.0%) | 14014 (6.5%) |
|  | Short (N=23,677) | 40473 (18.7%) | 1409 (0.7%) | 847 (0.4%) | 1265 (0.6%) | 8117 (3.8%) | 1594 (0.7%) | 639 (0.3%) | 9184 (4.2%) | 17419 (8.1%) | 16559 (7.7%) |
| **2017-2019** | Long (N=46,207) | 30344 (14.1%) | 387 (0.2%) | 852 (0.4%) | 1847 (0.9%) | 7370 (3.4%) | 2194 (1.0%) | 279 (0.1%) | 8573 (4.0%) | 8842 (4.1%) | 12450 (5.8%) |
|  | Middle (N=147,042) | 38310 (17.8%) | 555 (0.3%) | 687 (0.3%) | 1594 (0.7%) | 9104 (4.2%) | 1944 (0.9%) | 354 (0.2%) | 9809 (4.6%) | 14262 (6.6%) | 15740 (7.3%) |
|  | Short (N=21,804) | 41949 (19.5%) | 921 (0.4%) | 769 (0.4%) | 1370 (0.6%) | 9290 (4.3%) | 1444 (0.7%) | 434 (0.2%) | 9961 (4.6%) | 17761 (8.3%) | 16726 (7.8%) |
| **2020-2022** | Long (N=54,150) | 35284 (15.9%) | 408 (0.2%) | 764 (0.3%) | 2081 (0.9%) | 8224 (3.7%) | 2645 (1.2%) | 292 (0.1%) | 9423 (4.2%) | 11448 (5.2%) | 17134 (7.7%) |
|  | Middle (N=147,443) | 45502 (20.5%) | 576 (0.3%) | 800 (0.4%) | 1493 (0.7%) | 10524 (4.7%) | 2466 (1.1%) | 306 (0.1%) | 11828 (5.3%) | 17506 (7.9%) | 20783 (9.4%) |
|  | Short (N=20,289) | 46288 (20.9%) | 968 (0.4%) | 948 (0.4%) | 881 (0.4%) | 9749 (4.4%) | 1855 (0.8%) | 550 (0.2%) | 11149 (5.0%) | 20187 (9.1%) | 19401 (8.7%) |

*Mental disorder diagnoses reflect lifetime prevalence up to age 15.5, based on any recorded primary or secondary diagnosis. Diagnostic data are available from 1995 onward.

**Supplementary Figure 3:** Odds ratios (OR) of any mental disorder diagnoses from age 7-15.5 by a) income group and b) parents’ educational level.


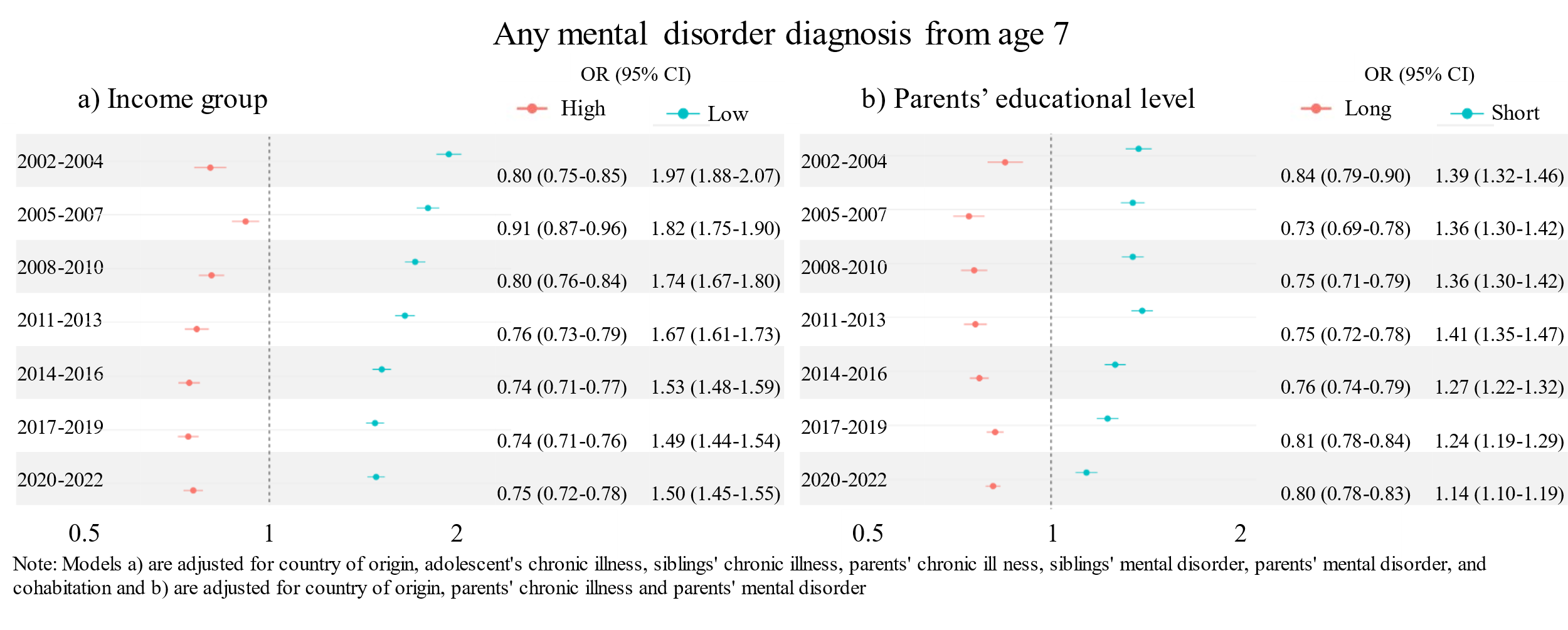


| **Supplementary Table 6: Mental disorder diagnoses from age 7 in 15-year-olds grouped in 3-year periods** | | | | | | | | |  |
| --- | --- | --- | --- | --- | --- | --- | --- | --- | --- |
| **Cohort** | | **2002-2004** | **2015-2007** | **2008-2010** | **2011-2013** | **2014-2016** | **2017-2019** | **2020-2022** | |
|  |  | N=189117 | N=208948 | N=221302 | N=215761 | N=216274 | N=215053 | N=221882 | |
| **Mental disorder diagnosis*** | Any mental disorder | 11468 (6.1%) | 15261 (7.3%) | 20154 (9.1%) | 23481 (10.9%) | 30224 (14.0%) | 33164 (15.5%) | 38652 (17.5%) | |
|  | Mean age of onset (95% CI) | 12.03 (11.98-12.09) | 12.05 (12.01-12.10) | 11.99 (11.95-12.03) | 11.86 (11.82-11.90) | 11.62 (11.59-11.66) | 11.52 (11.49-11.56) | 11.54 (11.51-11.58) | |
|  | Substance use disorders | 1387 (0.7%) | 1532 (0.7%) | 1529 (0.7%) | 847 (0.4%) | 685 (0.3%) | 501 (0.2%) | 501 (0.2%) | |
|  | Mean age of onset (95% CI) | 14.38 (14.33-14.43) | 14.45 (14.41-14.50) | 14.33 (14.29-14.38) | 14.39 (14.32-14.46) | 14.49 (14.42-14.56) | 14.48 (14.39-14.57) | 14.48 (14.40-14.56) | |
|  | Psychotic disorders | 254 (0.1%) | 332 (0.2%) | 411 (0.2%) | 529 (0.2%) | 660 (0.3%) | 722 (0.3%) | 795 (0.4%) | |
|  | Mean age of onset (95% CI) | 13.79 (13.57-14.00) | 13.59 (13.38-13.79) | 13.50 (13.31-13.68) | 13.79 (13.63-13.94) | 13.78 (13.66-13.91) | 13.67 (13.54-13.79) | 13.60 (13.47-13.73) | |
|  | Mood disorders | 552 (0.3%) | 784 (0.4%) | 1096 (0.5%) | 1344 (0.6%) | 1743 (0.8%) | 1616 (0.8%) | 1569 (0.7%) | |
|  | Mean age of onset (95% CI) | 14.04 (13.92-14.16) | 13.94 (13.84-14.05) | 14.03 (13.95-14.12) | 13.84 (13.76-13.92) | 13.74 (13.67-13.82) | 13.56 (13.48-13.65) | 13.77 (13.69-13.85) | |
|  | Anxiety-related disorders | 2210 (1.2%) | 3448 (1.7%) | 4256 (1.9%) | 5255 (2.4%) | 7100 (3.3%) | 8265 (3.9%) | 9194 (4.2%) | |
|  | Mean age of onset (95% CI) | 12.97 (12.88-13.06) | 13.13 (13.06-13.20) | 13.10 (13.04-13.16) | 13.09 (13.03-13.14) | 13.03 (12.98-13.08) | 12.80 (12.76-12.85) | 12.60 (12.55-12.64) | |
|  | Eating disorders | 577 (0.3%) | 722 (0.3%) | 870 (0.4%) | 1103 (0.5%) | 1383 (0.6%) | 1628 (0.8%) | 2066 (0.9%) | |
|  | Mean age of onset (95% CI) | 13.34 (13.19-13.50) | 13.06 (12.90-13.21) | 13.30 (13.17-13.43) | 13.50 (13.40-13.61) | 13.36 (13.27-13.46) | 13.17 (13.07-13.26) | 12.98 (12.89-13.07) | |
|  | Personality disorders | 375 (0.2%) | 360 (0.2%) | 381 (0.2%) | 346 (0.2%) | 417 (0.2%) | 325 (0.2%) | 311 (0.1%) | |
|  | Mean age of onset (95% CI) | 13.92 (13.74-14.11) | 13.65 (13.45-13.84) | 13.93 (13.75-14.12) | 14.01 (13.84-14.18) | 13.97 (13.81-14.14) | 13.88 (13.67-14.08) | 14.16 (13.98-14.34) | |
|  | Developmental disorders | 1974 (1.0%) | 2668 (1.3%) | 4022 (1.8%) | 5137 (2.4%) | 7075 (3.3%) | 8196 (3.8%) | 9627 (4.4%) | |
|  | Mean age of onset (95% CI) | 10.92 (10.82-11.03) | 10.97 (10.87-11.06) | 11.41 (11.33-11.49) | 11.60 (11.53-11.66) | 11.42 (11.36-11.48) | 11.47 (11.42-11.53) | 11.54 (11.49-11.59) | |
|  | Behavioral disorders | 4139 (2.2%) | 5415 (2.6%) | 7589 (3.4%) | 8920 (4.1%) | 11161 (5.2%) | 11911 (5.6%) | 14589 (6.6%) | |
|  | Mean age of onset (95% CI) | 11.01 (10.93-11.08) | 11.05 (10.98-11.12) | 11.29 (11.24-11.35) | 11.20 (11.15-11.25) | 10.92 (10.87-10.97) | 10.81 (10.77-10.86) | 11.07 (11.02-11.11) | |

*Mental disorder diagnoses reflect lifetime prevalence up to age 15.5, based on any recorded primary or secondary diagnosis. Diagnostic data are available from 1995 onward.
